# Effects of colloid pre-loading on thromboelastography during elective intracranial tumor surgery in pediatric patients: hydroxyethyl starch 130/0.4 versus 5% human albumin

**DOI:** 10.1186/s12871-017-0353-z

**Published:** 2017-04-27

**Authors:** Yuanzhi Peng, Jianer Du, Xuan Zhao, Xueyin Shi, Yingwei Wang

**Affiliations:** 1 Department of Anesthesiology, Xinhua Hospital Affiliated to Shanghai Jiao Tong, Shanghai, 200092 China; 2 Department of Anesthesiology, Huashan Hospital, FuDan University, Shanghai, 200040 China

**Keywords:** Hydroxyethyl starch 130/0.4, Thromboelastography, Pediatric, Intracranial tumor surgery

## Abstract

**Background:**

Volume replacement therapy with colloid is still worth studying in major pediatric surgery with potential risk of bleeding. This study assessed the effects of 6% hydroxyethyl starch (HES) 130/0.4 and 5% Human Albumin (HA) on coagulation tested by thromboelastography (TEG) during elective intracranial tumor surgery in pediatric patients.

**Methods:**

In this randomized controlled trial, 60 patients undergoing intracranial tumor resection under general anesthesia were assigned to HES and HA groups (*n* = 30), and administered preloads of 20 mL · kg^−1^ HES 130/0.4 and 5% HA, respectively, prior to dura opening. Primary outcomes were perioperative thromboelastography findings, and hemodynamic and hematological parameters. Blood transfusion, perioperative fluid balance, intracranial pressure, mortality, intensive care unit stay, and hospital stay were also assessed.

**Results:**

TEG parameters did not differ after preloading compared to baseline values in either group, except for a decrease in maximum amplitude immediately after infusion (HES group, 57.6 ± 6.0 mm vs. 50.9 ± 9.2 mm; HA group, 60.1 ± 7.9 mm vs. 56.6 ± 7.1 mm; *p* < 0.01), which was restored to preoperative levels 1 h after fluid infusion. Total perioperative fluid balance, blood loss or transfusion, intracranial pressure, and hematological and hemodynamic variables were similar between both groups (*p* > 0.05). Mortality, length of hospital stay, and clinical complications were similar between both groups.

**Conclusion:**

These findings suggest that HES and HA might have no significant differences regarding coagulation as assessed by TEG during pediatric intracranial tumor surgery with 20 ml/kg volume pre-loading, which can maintain stable hemodynamics and may represent a new avenue for volume therapy during brain tumor resection in pediatrics.

**Trial registration:**

ChiCTR-IPR-16009333, retrospectively registered October 8, 2016

## Background

Hypovolemia is the most common cause of circulatory failure in children during intracranial tumor surgery. Appropriate fluid replacement is essential for ensuring safety, and reducing morbidity and mortality in the perioperative period. Treatment options for replacing blood volume deficiencies in pediatric surgical patients include crystalloids and colloids. In case of acute and massive blood loss, colloids may be used to rapidly treat or prevent hypovolemia, due to prolonged intravascular half-life and improved intravascular volume effects [1, 2]. However, the ideal colloid therapy has not been found in pediatric patients undergoing intracranial tumor surgery.

Human albumin (HA) is frequently used for volume therapy in many centers during pediatric anesthesia. For many years, 5% HA was considered the gold standard for plasma volume replacement in infants and children due to physiological hypoproteinemia [1, 3], but its use is limited by high cost. Therefore, synthetic colloids are increasingly used, with each having unique physicochemical characteristics that determine the likely efficacy and adverse effects [4].

Hydroxyethyl starch (HES) is a plasma substitute widely used to correct perioperative hypovolemia. HES is a superior option over HA for its lower cost, so it has become the most commonly used artificial colloid in the world. The clinical efficacy of 6% HES 130/0.4 appears to be similar to that of other HES choices, with a significantly lower effect on patient coagulation parameters [5, 6], probably because of quicker achievement of optimal in vivo molecular weight and degree of molar substitution. It was reported that HES 130/0.4 may be associated with altered coagulation parameters in adult and pediatric cardiac surgery patients [7]; meanwhile, a recent systematic review reported that HES is not associated with increased blood loss or erythrocyte transfusion in surgical patients, particularly those undergoing cardiac surgery [8]. However, controlled studies assessing HES in children are scarce. Patients undergoing elective neurosurgical procedures sometimes require large volumes of intravenous fluid to maintain hemodynamic homeostasis during surgery. During pediatric intracranial tumor resection, the probability of massive hemorrhage is very high, making it necessary to expand blood volume for maintaining circulation stability. The main goal of intraoperative fluid management is to achieve hemodynamic homeostasis by the administration of small fluid volumes while avoiding fluid overload [9, 10].

The aim of this prospective, randomized, controlled study was to compare 6% HES 130/0.4 with 5% HA for volume replacement therapy during elective intracranial tumor resection in children aged 1–12 years. We tested the hypothesis that 6% HES 130/0.4 more effectively affects blood coagulation than 5% HA, as assessed by thromboelastography (TEG), during brain tumor resection in pediatric patients.

## Methods

In this prospective, randomized, double blind trial, 60 pediatric patients (aged 1–12 years) with American Society of Anesthesiologists physical status III, scheduled to undergo elective intracranial tumor resection at Xinhua Hospital affiliated to Shanghai Jiao Tong between October 2011 and January 2013, were sequentially enrolled. The study protocol was approved by the Institutional Ethical Committee of Xinhua Hospital. Parents or legal representatives were informed orally, and provided written consent before enrollment of the pediatric patients. Tumor locations included the saddle area, fourth and third ventricles, and other subcortical regions. Exclusion criteria were: known preoperative coagulation disorders, renal disease or plasma creatinine levels >1.5 mg/dL; known liver disease or increased plasma levels of alanine aminotransferase (>50 U · L^−1^) or aspartate aminotransferase (>50 U · L^−1^); administration of coumarin anticoagulants, non-steroidal anti-inflammatory agents, acetylsalicylic acid, or colloid management within the past 2 weeks. Patients with craniopharyngioma and hypothalamic tumors were also excluded.

All patients received standardized anesthetic management. General anesthesia was induced with intravenous atropine (0.01 mg · kg^−1^), midazolam (0.1 mg · kg^−1^), propofol (2–3 mg · kg^−1^), remifentanil (1–2 μg · kg^−1^), and cisatracurium (2 μg · kg^−1^). Anesthesia was maintained with continuous infusion of propofol (4–6 mg · kg^−1^h^−1^), remifentanil (0.05–0.1 μg · kg^−1^min^−1^), cisatracurium (1–2 μg · kg^−1^min^−1^), and sevoflurane (1.5–2%) in 2 L/min oxygen. Radial artery, great saphenous vein, and jugular vein catheters were inserted after endotracheal intubation. Intraoperative monitoring parameters consisted of end-tidal CO_2_, electrocardiographic measurements, central venous pressure, arterial blood pressure, pulse oximetry, and esophageal temperature. Mechanical ventilation was performed in all patients (50% air in oxygen) to maintain an O_2_ saturation of >95% and an end-expiratory CO_2_ of 25–30 mmHg. A warming air system and fluid warmer were used to avoid hypothermia during surgery. After surgery, mechanical ventilation was discontinued, and the patient was ready for tracheal extubation (stable hemodynamics, sufficient spontaneous breathing, temperature >36 °C). All patients were transferred to the intensive care unit (ICU) for postoperative treatment and patient-controlled analgesia.

Immediately after jugular vein catheter insertion, the patients were randomly divided to receive 20 mL · kg^−1^ each of 5% HA solution (Baxter Healthcare Corporation, Los Angeles, CA, USA) (HA group, *n* = 30) and a third-generation low-molecular HES solution (6% HES 130/0.4; Voluven; molecular weight 130 kDa, molar substitution ratio 0.4; Fresenius Kabi, Bad Homburg, Germany) (HES group, *n* = 30), respectively. Randomization was based on a computer generated code, and sealed in sequentially numbered, opaque envelopes. Both solutions were supplied in identical-looking, sequentially numbered plastic bags containing either 6% HES 130/0.4 or 5% HA according to the random code. Patients remained blinded throughout the study. Allocation to treatment groups was not known to any of the investigators, surgeons, or anesthetists. To compensate for fluid loss by sweating as well as through the gastric tube and urine, 10 mL · kg · h^−1^ lactated Ringer’s solution was administered to meet the basic requirements of the children. Throughout the study period, packed red blood cells (PRBC) were given when hemoglobin (Hb) was <8 g/dL; fresh frozen plasma (FFP) was administered with bleeding exceeding 50% of blood volume. When mean arterial blood pressure (MAP) was <50 mmHg despite sufficient central venous pressure (CVP >10 cmH_2_O), phenylephrine (1 μg/kg) was given. Epinephrine (0.03–0.1 μg/kg.min) was added when volume therapy and phenylephrine could not maintain the MAP above 50 mmHg, as a result of massive hemorrhage. Volume administration was completed in 1 h prior to dura opening. Hemodynamic measurements were recorded by invasive blood pressure monitoring. All patients were managed perioperatively by anesthetists not involved in the study and blinded to its aim.

Hemodynamic data (heart rate [HR], MAP, and CVP) were recorded after anesthesia induction (baseline), after volume infusion, 1 h after infusion, and at the end of surgery. Cranial window pressure was also recorded after fluid infusion, according to the surgeon’s level of experience (pressure was considered high, medium, and low according to brain tissue or meningeal bulging). Then, pH, Hb levels, hematocrit (Hct), base excess, and lactate in whole blood at different time points (baseline, after infusion, 1 h after infusion, at the end of surgery) were determined on a GEM Premier 3000 BLOOD Gas/Electrolyte Analyzer (IL company, Lexington, MA, USA). Estimated blood loss according to the suction apparatus, urine output, mannitol dosage (according to the surgeon’s request in case of increased intracranial pressure), and the amounts of blood products and lactated Ringer’s solution transfused were recorded at the end of surgery.

Four blood samples for TEG analysis were obtained from the jugular vein catheter at different time points: after anesthesia induction and before HES or HA infusion (baseline), immediately after infusion, 1 h after infusion and at the end of surgery. In order to avoid venous transfusion contamination, the blood sample collection method was standardized. All samples were obtained using the two-syringe method through the jugular vein catheter after first aspirating and discarding 5 mL of blood, a second 3-mL sample used for TEG measurements was collected and transferred into blue-capped non-wettable surface tubes containing 0.2 mL of 3.2% sodium citrate. The tubes were capped, with the blood mixed with citrate three times by gentle inversion. All blood samples were analyzed at 37 °C using disposable plastic cups and pins on a computerized Thrombelastograph Coagulation Analyzer (Model 3000; Haemoscope, IL, Lexington, USA). A 360-μL aliquot was pipetted and analyzed 4 min after sample collection. The TEG analyzer was allowed to run until LY60 (reduction in the maximal amplitude of the TEG tracing after 60 min) value could be completed. Four parameters of clot formation were measured on the TEG analyzer, including r-time (reaction time to clot formation), k-time (time to achieve clot strength), MA (maximal amplitude of clot strength)and the α-angle (angle of divergence). The coagulation index (CI)of TEG derived from a linear equation that combines the four variables of TEG (based on Haemoscope Corporation as printed in the Thrombelastograph Coagulation Analyzer 3000 manual; CI = −0.6516r - 0.3772 k + 0.1224MA + 0.0759α - 7.792) was also calculated.

### Statistical analysis

The sample size was estimated with two-sided α and β errors of 0.05 and 0.2, respectively. A 20% decrease in the MA of the TEG tracing (i.e., strength of the fibrin clot) with HES treatment was assumed [11]; the power analysis indicated that a minimum sample size of 30 patients was required for each group.

All measured and calculated data were analyzed using SPSS 16.0 (SPSS, Chicago, IL, USA), and expressed as mean ± SD or number of patients. Normality was verified using the Kolmogorov-Smirnov test. Data were assessed by unpaired Student’s *t*-test, chi-square test, or Fisher’s exact test, as appropriate. For intragroup comparisons of variables to baseline values, repeated measures analysis of variance, followed by a post hoc Dunnett’s test were used. *P* < 0.05 was considered statistically significant.

## Results

The 60 enrolled patients all completed the study. Patient demographic and biometric data, and anesthesia and surgery durations did not differ between groups (Table 1).

**Table 1:** Demographic characteristics and operative data

	HES	HA
Gender(M/F)	8/22	9/21
Age(yr)	4.5 ± 3.3	4.3 ± 3.4
Weight(kg)	21.9 ± 10.9	19.1 ± 10.0
Surgery duration (h)	3.1 ± 0.9	3.7 ± 1.0
Anesthesia duration (h)	4.2 ± 1.1	4.6 ± 1.2
Type of surgery		
Saddle area	9	11
The fourth ventricle	10	9
The third ventricle	5	6
Other subcortical	6	4

Data are mean ± SD or number of patients. HES: 6% hydroxyethyl starch130/0.4; HA: 5% human albumin

**Fig. 1:**
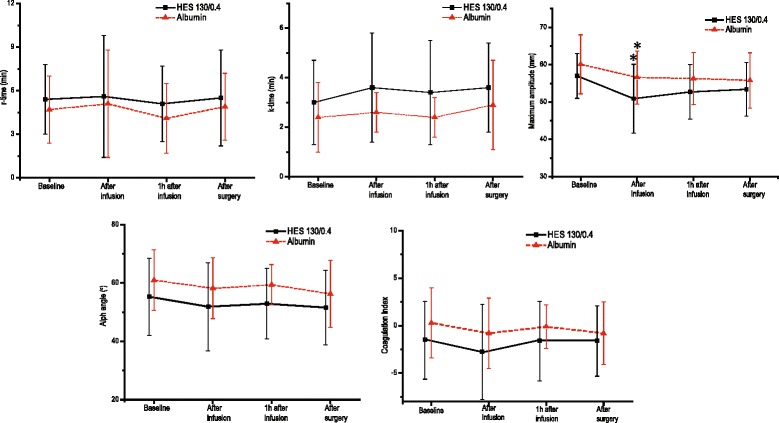
Thromboelastographic data before and after colloid preloading in pediatric patients receiving 6% hydroxyethyl starch 130/0.4 and 5% human albumin, respectively. Data are mean ± standard deviation (SD). *P < 0.01 vs. baseline data. HES, 6% hydroxyethyl starch 130/0.4; HA, 5% human albumin; r-time, reaction time (normal range, 5–10 min); k-time, clot formation time (normal range, 1–3 min); α-angle, clot formation rate (normal range, 53°–72°); maximum amplitude (normal range, 50–70 mm); coagulation index (normal range, −3–3)

There were no statistical significant differences in TEG variables between groups throughout the study period. Initial reaction time (r-time), clot formation time (k-time), speed of solid clot formation (α-angle), and CI were similar to baseline values after preloading fluid infusion (Fig. 1). In both groups, only fibrin clot strength (MA) was decreased after preloading fluid infusion compared with baseline values (HES group, 57.6 ± 6.0 mm vs. 50.9 ± 9.2 mm; HA group, 60.1 ± 7.9 mm vs. 56.6 ± 7.1 mm; *P* < 0.01 for both groups); however MA was restored to the preoperative level at 1 h after fluid infusion (HES group, 52.7 ± 7.3 mm; HA group, 57.1 ± 7.1 mm; Fig. 1).

No measured hemodynamic parameters (MAP, HR) differed significantly between the two groups within the study period except for CVP, which was elevated after preloading fluid infusion compared with baseline values (HES group, 5.2 ± 1.7 vs. 9.7 ± 2.4 cm H_2_O; HA group, 5.4 ± 1.6 vs. 7.9 ± 1.9 cm H_2_O; *P* < 0.01 for both groups). Perioperative changes were observed in Hb levels (HES group, 12.3 ± 1.3 vs. 9.9 ± 1.1 g/dL; HA group, 11.8 ± 1.4 vs. 9.8 ± 1.3 g/dL; *P* < 0.01 for both groups) and Hct (HES group, 37.4 ± 4.2 vs. 29 ± 3.4; HA group, 36.5 ± 4.7 vs. 29.3 ± 4.2; *P* < 0.01 for both groups) after preloading fluid infusion, but not in PH, lactate, or base excess (Table 2). There were no intergroup differences in intraoperative blood loss, amounts of infused crystalloids and mannitol to decrease intracranial pressure or urine output during surgery, and requirements for transfusion with PRBC or FFP (Table 3).

**Table 2:** Hematological, hemodynamic data, and blood gas analysis

Variable	group	T0	T1	T2	T3
Hb(g/dL)	HES	12.3 ± 1.3	9.9 ± 1.1^*^	10.3 ± 1.5	10.7 ± 1.6
	HA	11.8 ± 1.4	9.8 ± 1.3^*^	10.5 ± 0.8	10.4 ± 1.0
Hematocrit (%)	HES	37.4 ± 4.2	29 ± 3.4^*^	27.8 ± 4.8	29.3 ± 5.1
	HA	36.5 ± 4.7	29.3 ± 4.2^*^	28.7 ± 5.4	29.3 ± 3.3
Lactate (mmol/L)	HES	1.5 ± 0.8	1.7 ± 1.0	2.0 ± 1.3	2.5 ± 1.7
	HA	1.6 ± 1.1	1.6 ± 0.8	2.1 ± 1.4	2.0 ± 1.3
BE(mmol/L)	HES	1.2 ± 2.3	−1.2 ± 2.6	−0.5 ± 2.8	−0.3 ± 2.6
	HA	1.7 ± 2.4	1.5 ± 2.2	0.7 ± 3.0	1.0 ± 3.0
PCO_2_(mm Hg)	HES	35.2 ± 5.2	31.4 ± 3.6	30.7 ± 4.5	38.7 ± 5.8
	HA	33.1 ± 4.7	30.5 ± 3.3	31.1 ± 4.9	39.2 ± 5.5
PH	HES	7.46 ± 0.06	7.45 ± 0.06	7.46 ± 0.05	7.43 ± 0.06
	HA	7.47 ± 0.05	7.47 ± 0.07	7.47 ± 0.07	7.44 ± 0.07
HR(b/min)	HES	105 ± 15	95 ± 15	95 ± 13	100 ± 17
	HA	105 ± 18	100 ± 16	102 ± 18	102 ± 18
MAP(mm Hg)	HES	72.7 ± 9.4	67.7 ± 7.9	67.3 ± 9.1	71.8 ± 10.7
	HA	72.1 ± 9.2	66.4 ± 8.6	66.4 ± 7.7	69.7 ± 9.2
CVP(cmH_2_O)	HES	5.2 ± 1.7	9.7 ± 2.4^*^	7.4 ± 2.4	6.9 ± 2.1
	HA	5.4 ± 1.6	7.9 ± 1.9^*^	7.9 ± 1.9	7.9 ± 2.0

Data are mean ± SD. HES: 6% hydroxyethyl starch130/0.4; HA: 5% human albumin. T0: Baseline, T1: After infusion, T2: 1 h after infusion, T3: at the end of surgery. *p < 0.05 compared with baseline data

**Table 3:** Fluid in- and output and use of blood products during surgery

	HES	HA
Colloids (ml/kg)	20	20
Crystalloids(ml)	545 ± 224	565 ± 230
Mannitol(ml)	148 ± 63	113 ± 50
Red blood cells(unit)	2.4 ± 1.2	2.8 ± 1.4
Fresh frozen plasma(ml)	224 ± 159	216 ± 130
Blood loss(ml)	356 ± 308	391 ± 337
Urine output(ml)	720 ± 490	558 ± 368

Data are mean ± SD. HES: 6% hydroxyethyl starch130/0.4; HA:5% human albumin

Postoperative complications, including infection, diabetes insipidus, and respiratory complications did not differ between the two groups; however, one patient in the HES group died from cerebral hernia and respiratory failure. No intergroup differences were obtained in ICU duration (HES group, 3.1 ± 1.2 vs. HA group, 3.8 ± 1.6 days) and hospital stay (HES group, 19.0 ± 8.3 vs. HA group, 22.2 ± 11.0 days). The cranial window pressure increased moderately in both groups after preloading infusion (HES group, 20 cases; HA group, 22 cases; *P* = 0.57), but no significant difference was found in mannitol dosage between the two groups (Table 3).

## Discussion

This study assessed the effects of HES 130/0.4 as a perioperative volume substitution compared to 5% HA in children <12 years of age. This is the first clinical trial evaluating the effects of a high-dose HES preparation on coagulation in children undergoing intracranial tumor resection. The major finding of this study was that volume expansion and hypervolemia induced with HES and 5% HA following anesthesia induction in pediatric intracranial tumor surgery had similar effects on TEG parameters. No significant differences were observed between the two colloids with regard to intraoperative blood loss, transfusion requirements, hemodynamic stability, and postoperative complications.

Among the various colloids, HES solutions have been used as clinical expansion agents for mang years. However, our investigation is the only study to compare large-dose HES 130/0.4 with HA in pediatric patients undergoing intracranial tumor surgery, while equivalent volume expansion capacity were shown by HES 130/0.4 and 5% HA in this study. High dose of HES solutions may impair coagulation, the compounds with higher molecular weights and greater molar substitutions having more pronounced effects than those with lower molecular weights or fewer substitutions [12].

HES130/0.4 has been widely used for volume replacement therapy in Europe since its approval in 1999/2000, where its currently approved daily maximum dose is 50 mL/kg body weight in adults. In patients with severe traumatic brain injury, it causes no coagulopathy or bleeding complications even at higher doses up to 70 mL/kg [13]. Similar results were also observed in heart disease patients with infusions of as high as 50 mL/kg HES 130/0.4 [14]. This is the first study to investigate the effect of HES 130/0.4 with 20 ml/kg volume pre-loading on the coagulation function in children with brain tumors, the TEG results showed that HES 130/0.4 did not increase the coagulation disorder compared with 5% HA. HES 130/0.4 has little effect on the coagulation function compared with other HES solutions, which has been confirmed by in vitro experiment [15]. As Sudfeld retrospective study showed, the kidney function tested by cystatin C-derived estimated glomerular filtration rates, the renal function was only mildly affected by administration of a median dose of 1000 mL of 6% HES 130/0.4 in patients undergoing radical prostatectomy, which indicates that HES 130/0.4 does not exert harmful effects on renal function [16]. The main adverse effect of HES solutions is impaired coagulation as demonstrated by reduced von Willebrand factor (vWF) antigen concentration and factor VIII activity [17]. In this study, single factors of the coagulation system (e.g., vWf) were not assessed because they have only limited importance when assessing the effects of different intravascular volume replacement regimens on the coagulation process. Thus, TEG measurements have become an accepted measure to assess coagulation changes after intravascular volume replacement [18].

A recent study comparing the novel 6% HES 130/0.4 with 4% HA in 119 children undergoing cardiac surgery with cardiopulmonary bypass reported equal blood losses with the priming solutions, but an increased need for allogenic blood transfusion in the HA group [19]. In blood samples, HES binds in a concentration-dependent manner to the platelet surface, and is believed to cause platelet aggregation inhibition [20, 21]. HES 130/0.4 has a lesser effect on platelet function compared with other HES solutions, including HES 450/0.7, HES 70/0.5 and HES 200/0.5 [22]. The less antiplatelet effect of HES 130/0.4 may be due to fewer substitutions in the HES molecule. Although platelet function was not examined in the current study, the reduced effect on platelets may have contributed to the absence of serious coagulopathies.

Decreased Hb levels and increased CVP after HES infusion indicates effective plasma expansion. In the current study, Hb amounts and Hct decreased mildly, and CVP increased accordingly in both groups, indicating effective plasma expansion. Although intracranial pressure increased moderately in most patients, volume expansion was not the only influencing factor; rather, tumor size and location also had an effect.

Safety information about the new HES preparation, especially pertaining to detailed coagulation data in pediatric patients, is limited. Using progressive in vitro hemodilutions (30% and 60%), Jamnicki [23] assessed the effects of different HES preparations (HES 200/0.5 and HES 130/0.4) on conventional TEG data, and found that the two solutions affect in vitro coagulation to the same degree (r and k increased; MA and α angle decreased progressively). However, Lochbühler [24] found no clinically significant changes in platelet count, prothrombin time, and activated partial thromboplastin time after HES 130/0.4 infusion in neonates and infants. Chong Sung [25] showed that administration of 10 mL · kg^−1^ HES 130/0.4 to children undergoing cardiac surgery does not cause more bleeding or increase transfusion requirements compared with FFP infusion. Haas et al.[3] demonstrated that activated modified TEG values are significantly more impaired after HES 130/0.4 infusion than after HA or gelatin infusion, using a high infusion volume of 15 mL · kg^−1^ in children weighing 3–15 kg. A recent systematic review [26] in Europe showed that moderate doses of HES 130/0.42 for perioperative plasma volume replacement in children cause only moderate changes in Hb levels and acid-base balance, suggesting that this compound is safe and effective even in neonates and young infants with normal renal function and coagulation ability. These findings corroborate previous studies demonstrating that HES can be used safely even in young children with an intact coagulation system [3, 19, 26].

A major limitation of this study is that only changes of short-term coagulation function during the perioperative period were evaluated. Although the MA value can partly reflect fibrinogen activity and platelet function, we did not assess the effects of shear forces on the interactions between coagulation factors and platelet function [27]. In this study, brain tissue or meningeal bulging was used as an alternative tool to indirectly monitor intracranial hypertension. We found that intracranial pressure increased moderately in both groups, and may also be affected by the size and location of brain tumors. In clinical practice, intracranial pressure is not measured routinely during elective neurosurgical procedures, which is an additional limitation of the present study. However, volume preloading may leave patients with an excessive fluid load that increases the risks for various complications, such as intracranial hypertension or even cerebral edema, which is also detrimental during the neurosurgical procedure performed in this study.

## Conclusion

In conclusion, the current study demonstrated that HES 130/0.4 and HA 20 ml/kg volume preloading appear to show no significant differences in coagulation parameters as assessed by TEG during pediatric intracranial tumor surgery. Blood loss or blood products used, hemodynamic changes, and clinical outcomes were also similar, suggesting that HES 130/0.4 can be safely used during intracranial tumor resection in pediatric patients. These findings indicate that HES 130/0.4 preloading may represent a new avenue for volume therapy during brain tumor resection in pediatrics.
